# *Diphyllobothrium sprakeri* n. sp. (Cestoda: Diphyllobothriidae): a hidden broad tapeworm from sea lions off North and South America

**DOI:** 10.1186/s13071-021-04661-1

**Published:** 2021-04-22

**Authors:** Jesús S. Hernández-Orts, Tetiana A. Kuzmina, Luis A. Gomez-Puerta, Roman Kuchta

**Affiliations:** 1 Institute of Parasitology, Biology Centre, Czech Academy of Sciences, Branišovská 31, 37005 České Budějovice, Czech Republic; 2 I. I. Schmalhausen Institute of Zoology NAS of Ukraine, 15, Bogdan Khmelnytsky Street, Kyiv, 01030 Ukraine; 3 Department of Veterinary Epidemiology and Economics, School of Veterinary Medicine, National University of San Marcos, Av. Circunvalación 2800, San Borja, 41 Lima, Peru

**Keywords:** Parasites, Helminths, *cox*1, *lsr*DNA, Pinnipedia, Otariidae, *Zalophus californianus*, *Otaria flavescens*

## Abstract

**Background:**

The systematic of several marine diphyllobothriid tapeworms of pinnipeds has been revised in recent years. However, 20 species of *Diphyllobothrium* from phocids and otariids are still recognized as *incertae sedis*. We describe a new species of *Diphyllobothrium* from the intestine of California sea lions *Zalophus californianus* (Lesson) (type-host) and South American sea lions *Otaria flavescens* (Shaw).

**Methods:**

*Zalophus californianus* from the Pacific coast of the USA and *O. flavescens* from Peru and Argentina were screened for parasites. Partial fragments of the large ribosomal subunit gene (*lsr*DNA) and the cytochrome *c* oxidase subunit 1 (*cox*1) mitochondrial gene were amplified for 22 isolates. Properly fixed material from California sea lions was examined using light and scanning electron microscopy.

**Results:**

A total of four *lsr*DNA and 21 *cox*1 sequences were generated and aligned with published sequences of other diphyllobothriid taxa. Based on *cox*1 sequences, four diphyllobothriid tapeworms from *O. flavescens* in Peru were found to be conspecific with *Adenocephalus pacificus* Nybelin, 1931. The other newly generated sequences fall into a well-supported clade with sequences of a putative new species previously identified as *Diphyllobothrium* sp. 1. from *Z. californianus* and *O. flavescens*. A new species, *Diphyllobothrium sprakeri* n. sp., is proposed for tapeworms of this clade.

**Conclusions:**

*Diphyllobothrium sprakeri* n. sp. is the first diphyllobothriid species described from *Z. californianus* from the Pacific coast of North America, but *O. flavescens* from Argentina, Chile and Peru was confirmed as an additional host. The present study molecularly confirmed the first coinfection of two diphyllobothriid species in sea lions from the Southern Hemisphere.
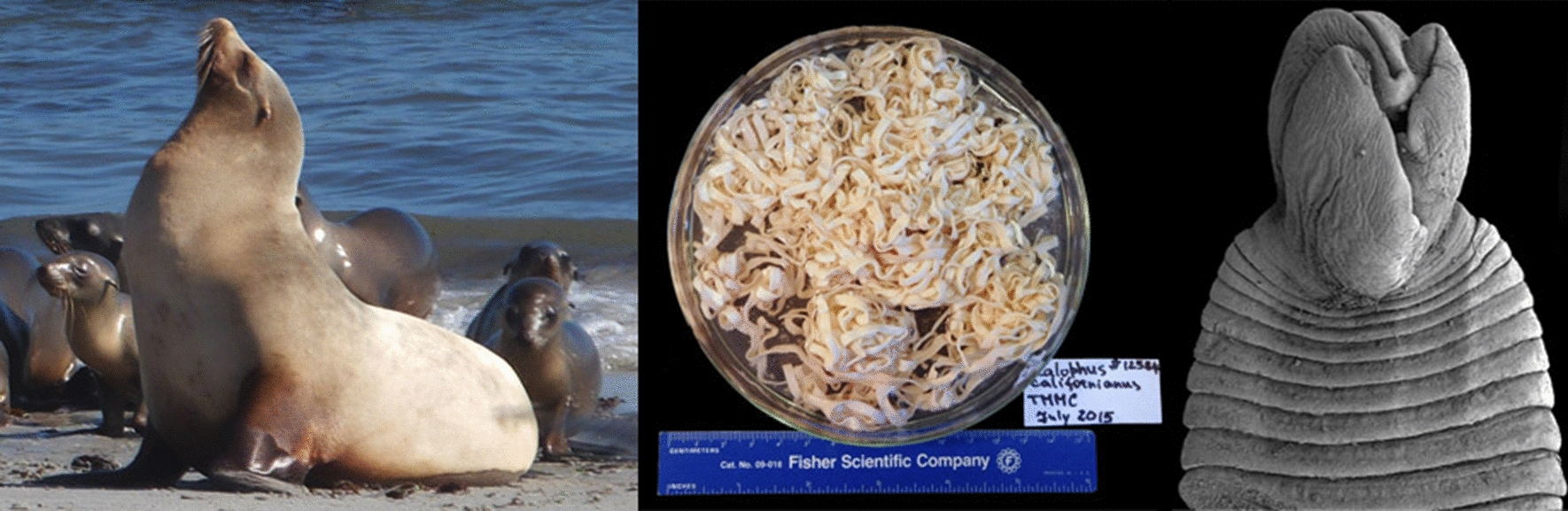

**Supplementary Information:**

The online version contains supplementary material available at 10.1186/s13071-021-04661-1.

## Background

The systematic of the tapeworms (Cestoda) has been under detailed revision since the last decade including adding new molecular data [1]. However, current knowledge and systematics of the tapeworms of marine mammals are still superficial [2–7]. The pinnipeds are well-known hosts of several groups of tapeworms composed of two orders, Tetrabothriidea with only seven members of the genus *Anophryocephalus* Baylis, 1922, and Diphyllobothriidea with around 30 species of several genera [7, 8]. Waeschenbach et al. [4] revised the systematic of diphyllobothriids based on morphological as well as molecular data and proposed systematic changes mainly in the polyphyletic genus *Diphyllobothrium* Cobbold, 1858, which now includes only seven species parasitizing cetaceans. Non-monophyletic *Diphyllobothrium* (designated as ‘*Diphyllobothrium*’ by Waeschenbach et al. [4])  provisionally comprises 20 species mainly from pinnipeds (eight of which have been characterized molecularly) that do not form a monophyletic assemblage and are considered *incertae sedis* [4].

The California sea lion *Zalophus californianus* (Lesson) (CSL) is a well-known otariid species of North America whose range in the Pacific is from Baja California, Mexico, to British Columbia, Canada. Nowadays, its population is increasing and includes around 340,000 individuals [9, 10]. The first diphyllobothriid tapeworm from CSL was reported by Stunkard [11] who studied a scolex of '*Diphyllobothrium*' sp. (see fig. 2 in Stunkard [11]). Later, several authors reported diphyllobothriids from CSL as *Adenocephalus pacificus* Nybelin, 1931 (under the names *D. glaciale* [Cholodkovsky, 1915] or *D. pacificum* [Nybelin, 1931]) [12–17]. The recent revision of metazoan parasites of CSL considered 24 valid species, including two undescribed tapeworm species [18].

The South American sea lion *Otaria flavescens* (Shaw) (SASL) is distributed exclusively in South America from the Pacific coast of Peru to the Atlantic coast of southern Brazil [19]. Nowadays, its population has been estimated as at least 445,000 individuals [20]. The first diphyllobothriid tapeworm from SASL was reported by Baylis [21] from Falkland Island (Islas Malvinas) as *Diphyllobothrium scoticum* (Rennie & Reid, 1912). This material was later studied by Markowski [22] who confirmed Baylis’ specimens as *D. scoticum*. However, the evaluation Baylis’ [21] material by Baer et al. [23] and Hernández-Orts et al. [2] suggested that these specimens belong to *A. pacificus*. Hermosilla et al. [24] reported a ‘*Diphyllobothrium scoticum*-like cestode’ from SASLs from the Pacific coast of Chile to be molecularly conspecific with samples from CSLs. The recent revision of metazoan parasites of SASL by Ebmer et al. [25] reported 44 metazoan taxa (only 25 were identified to the species level), including *A. pacificus* with its synonyms, *Clistobothrium  delphini* (Bosc, 1802) [as *Phyllobothrium delphini* (Bosc, 1802)] and unidentified species of *Anophryocephalus* Baylis, 1922 and '*Diphyllobothrium*'.

A detailed study of newly obtained material, examination of museum specimens, as well as molecular phylogenetic analysis and extensive literature revision confirmed the presence of an undescribed species of the genus *Diphyllobothrium* which parasitizes CSLs. The main goal of this article is the description of a new species previously reported as ‘*Diphyllobothrium*’ sp. 1 by Waeschenbach et al. [4], Hermosilla et al. [24] and Kuzmina et al. [18] on the basis of detailed morphological and molecular data.

## Methods

### Sample collection

A total of 39 CSLs, 9–10 months to 16 years old, were collected stranded on the Pacific coast of central California (36°57′–38°32′N, 121°95–123°00′W), USA, between 2012 and 2018 (see [18] for details). CSLs died in the Marine Mammal Center (Sausalito, California, USA) from different causes and were necropsied using a standard procedure [26]. Two recently dead SASLs were collected from two localities in South America: (i) a subadult male from Bellavista beach (12°04′S, 77°07′W), Callao City, Callao, Peru, in October 2017; (ii) a subadult female from Playa Unión (43°19′S, 65°03′W), Chubut, Argentina, in October 2013. The intestines of fresh SASLs were excised from the carcasses, opened and washed with tap water through a series of sieves. Intestinal contents were placed in Petri dishes with saline and examined under a dissecting microscope. Tapeworms were washed in saline and killed with hot (90 °C) tap water and fixed in 70% ethanol. A few posterior proglottids of selected specimens were cut off and fixed in molecular-grade ethanol (99%) for DNA sequencing before killing the worm.

### Molecular data and phylogenetic analyses

Three diphyllobothriid specimens from CSLs and 18 from SASLs (17 from Peru and one from Argentina) were selected for molecular studies. Pieces of strobila were used for DNA isolation and sequencing. The remaining parts of the worms were stained and mounted in Canada balsam and were kept as molecular vouchers (i.e. hologenophores sensu Pleijel et al. [27]). Total genomic DNA was extracted using DNeasy Blood and Tissue Kit (Qiagen, Hilden, Germany) according to the manufacturer’s protocol. Phylogenetic relationships of the studied diphyllobothriids were evaluated based on two molecular markers: the large subunit nuclear ribosomal RNA gene (*lsr*DNA) and the cytochrome *c* oxidase subunit 1 (*cox*1) mitochondrial gene.

Partial *lsr*DNA (D1–D3 domains, *ca.* 1400 bp) sequences were generated using the primers LSU5 (5′-TAG GTC GAC CCG CTG AAY TTA AGC A-3′; [28]) and 1500R (5′-GCT ATC CTG AGG GAA ACT TCG-3′; [29]). Partial (*ca.* 420 bp) and almost complete (*ca.* 1500 bp) *cox*1 sequences were amplified using the primers JB3 (5′-TTT TTT GGG CAT CCT GAC GTT TAT-3′; [30]) and JB 4,5 (5′-TAA AGA AAG AAC ATA ATG AAA ATG-3′ [30]) or the primers Cox1Forward (5′-TAT CAA ATT AAG TTA AGT AGA CTA-3′; [31]) and Cox1Reverse (5′-CCA AAT AGC ATG ATG CAA AAG-3′; [31]), respectively. PCR amplification reactions were performed following the procedures described by Brabec et al. [32] for the *lsr*DNA gene and Wicht et al. [31] or Gomez-Puerta et al. [33] for the *cox*1 gene. All products were purified through an enzymatic treatment with Exonuclease I and FastAP alkaline phosphatase (Thermo Fisher Scientific, Waltham, MA, USA) or using a Microcon® Centrifugal Filters (Millipore, Bedford, MA, USA). Purified products were Sanger sequenced at GATC Biotech (Konstanz, Germany) or using BigDye™ Terminator Cycle Sequencing Ready Reaction Kit (Applied Biosystems, Foster City, CA, USA) and an ABI 3100 automated sequencer (Applied Biosystems).

Sequences were assembled and inspected for errors using Geneious v.11 and deposited in the GenBank database (accession numbers MW600336–MW600339 for the *lsr*DNA sequences and MW596661–MW596682 for the *cox*1 sequences). Newly generated sequences were aligned in two independent datasets following the alignments from Hernández-Orts et al. [2] and Waeschenbach et al. [4]. Sequences from other diphyllobothriids were retrieved from GenBank and aligned with our novel sequences using default settings of MUSCLE [34] implemented in Geneious (Additional file 1: Table S1). The extremes were trimmed resulting in an alignment with 1574 bp for the *lsr*DNA (Additional file 2: *lsr*DNA_alignment) and 1571 bp for the *cox*1 (Additional file 3: *cox*1_alignment). A combined *lsr*DNA + *cox*1 alignment (3145 bp; Additional file 4: *lsr*DNA_*cox*1_alignment) was also constructed using only taxa with sequences for both markers available from GenBank (Additional file 1: Table S1).

Bayesian inference (BI) and maximum likelihood (ML) analyses were performed for each dataset. jModelTest 2.1.10 software [35] was used to select the best nucleotide substitution model under the Akaike information criterion. The TIM2 + I + G model was chosen for the *lsr*DNA and *cox*1 datasets and the TIM2 + I + G & TIM1 + I + G for the combined dataset. Bayesian inference analyses were constructed using MrBayes 3.2.6 [36]. The BI analyses were estimated via two independent Markov Chain Monte Carlo runs of four chains with standard settings for 10,000,000 generations with a sampling frequency of 1000th generations. Burn-in periods were set to 25% of generations. The ML analyses were run with raxmlGUI v.2.0 [37]. Bootstrap nodal support values were computed by running 1000 bootstrap resamples. The resulting trees for BL and ML were visualized in FigTree 1.4.4 [38]. Genetic distances (uncorrected *p*-distance) were calculated with MEGA 10.1.8 [39] from the total number of nucleotide differences from the *lsr*DNA alignment and from the full *cox*1 alignment excluding partially characterized sequences (i.e. < 1400 bp).

### Morphological examination

For morphological evaluation, selected tapeworms were stained with Mayer’s hydrochloric carmine, dehydrated through an ethanol series, cleared with eugenol and mounted in permanent slides in Canada balsam. Selected pieces of the strobila were embedded in paraffin wax, cross-sectioned (thickness 15 µm), stained with hematoxylin–eosin, and mounted in Canada balsam. Mounted specimens were examined with an Olympus BX51 microscope (Olympus Corp., Tokyo, Japan). Measurements were taken from digital images with the QuickPHOTO CAMERA 3.2 image analysis software (Promicra Ltd., Prague, Czech Republic). Measurements are expressed in micrometers unless otherwise stated and presented as the range (minimum–maximum), with the mean followed by the standard deviation (SD) and the number of measured specimens or structures in parentheses. Detailed line drawings were made using a drawing tube attached to an Olympus BX51 microscope.

Selected scoleces and proglottids were prepared for scanning electron microscopy (SEM). Specimens were dehydrated through an ethanol series, transferred to hexamethyldisilazane (Ted Pella, Inc., Redding, CA, USA) and allowed to air dry. Samples were mounted on aluminium stubs on double-sided adhesive carbon tape, gold sputter-coated and examined with a JEOL JSM 7401-F scanning electron microscope (JEOL Ltd., Tokyo, Japan) at 4 kV at the Laboratory of Electron Microscopy, Institute of Parasitology, Biology Centre, Czech Academy of Sciences. Terminology of microtriches follows Chervy [40].

Tapeworms collected in the present study were compared with voucher material of the following immature diphyllobothriid species collected from *Z. californianus* identified as '*Diphyllobothrium latum* (L.)' and deposited at the Natural History Museum (NHML), London, UK: two vouchers from San Diego County (NHML 1980.6.3.196–8), one from Los Angeles (NHML 1980.6.3.200), one from an unknown locality in California, USA (NHML 1994.7.21.12–13), and one from Mexican waters (NHML 1980.6.3.189).

Specimens of the type series and voucher specimens from the present study are deposited in the Helminthological Collection of the Institute of Parasitology (IPCAS), Biology Centre, Czech Academy of Sciences, České Budějovice, Czech Republic; the National Museum of Natural History of the Smithsonian Institution (NMNH-USNM), Washington, DC, USA; the Parasite Collection of the Museum of Natural History (PCMNH), National University of San Marcos, Lima, Peru; and the Collection of the Laboratory of Epidemiology and Veterinary Economics (LEVE), School of Veterinary Medicine, National University of San Marcos, Lima, Peru.

## Results

More than 150 tapeworm specimens (including immature and gravid specimens) were collected in the intestine of CSLs. In the SASLs from Argentina and Peru, 13 and 18 immature tapeworms were collected, respectively.

### Phylogenetic relationships and genetic divergence

A total of four partial *lsr*DNA sequences (1450–1481 bp long; three isolates from CSLs, USA, and an isolate from SASL, Argentina) were generated. Additionally, one almost complete (1565 bp long; isolate from SASL, Argentina) and 21 partial *cox*1 sequences (415–420 bp long; four isolates from CSLs, USA, and 17 isolates from SASL, Peru) were generated (see Additional file 1: Table S1).

**Fig. 1:**
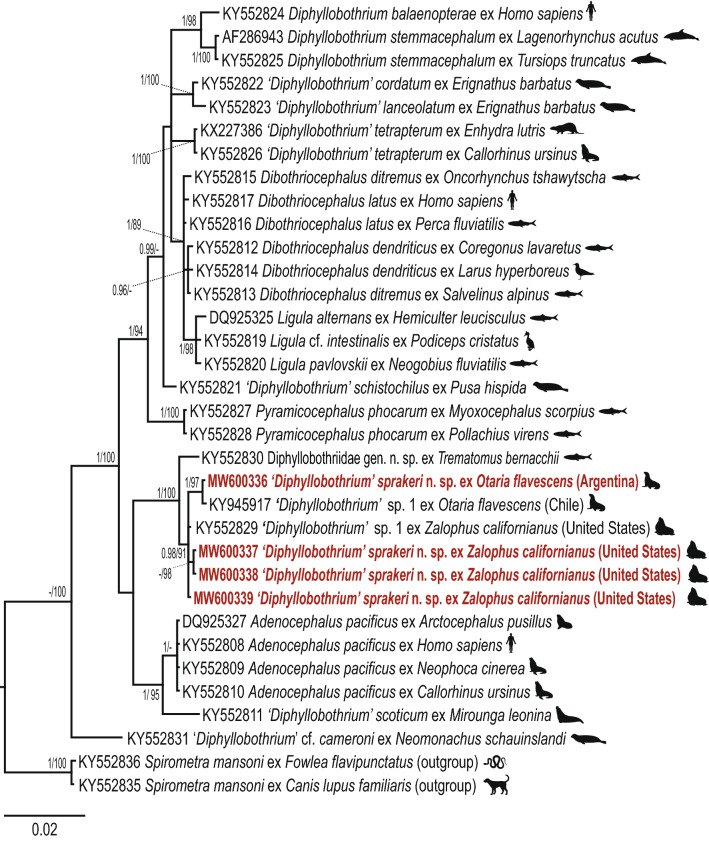
Bayesian analysis for the partial *lsr*DNA dataset for selected representatives of the family Diphyllobothriidae. Numbers represent posterior probabilities from BI analysis (> 0.95 shown only) followed by nodal supports from ML analysis (bootstrap values > 70% shown only). The newly generated sequences are indicated in red. GenBank accession numbers are shown before the species names. The scale bar indicates the expected number of substitutions per site

The resulting phylograms inferred with BI and ML analyses for the *lsr*DNA dataset (34 taxa) showed generally similar topologies (Fig. 1) and congruent results with those of Waeschenbach et al. [4]. Our four newly generated sequences formed a well-supported clade with sequences of a putative new species *‘Diphyllobothrium’* sp. 1 from CSL of California (KY552829) [4] and *‘Diphyllobothrium’* sp. 1 from SASL of Chile (KY945917) [19] (Fig. 1). The clade composed by our novel sequences and '*Diphyllobothrium*' sp. 1 appeared to be sister to a sequence generated from a diphyllobothriid plerocercoid (reported as Diphyllobothriidae gen. sp.) from *Trematomus bernacchii* Boulenger of Antarctica (KY552830) [4] (Fig. 1). '*Diphyllobothrium*' sp. 1 and Diphyllobothriidae gen. sp. form the sister group of a clade which includes *‘Diphyllobothrium’* *scoticum* from *Mirounga leonina* (L.) and *A. pacificus* isolates, but these two lineages with low support (Fig. 1).

Intraspecific sequence variability of 0.14% was only detected between the four isolates from CSLs and the two from SASLs for the *lsr*DNA gene. The *lsr*DNA sequences of isolates from CSLs and SASL diverged 0.64–0.78% from the sequences of Diphyllobothriidae gen. sp. from *T. bernacchii* from Antarctica. Genetic divergence values between our lineage and *‘Diphyllobothrium’ scoticum* were 2.48–2.55% and between *A. pacificus* ranged from 1.99–2.06%. Intergeneric distance between *'Diphyllobothrium' scoticum* and *A. pacificus* was 1.13%. The interspecific distances in the *lsr*DNA region of *'Diphyllobothrium'* spp. of pinnipeds range between 0.36% (*‘Diphyllobothrium’ cordatum* (Leuckart, 1863) *vs* *‘Diphyllobothrium’ lanceolatum* (Krabbe, 1865)) to 3.70% (*‘Diphyllobothrium’ tetrapterum* (von Siebold, 1848) *vs* *‘Diphyllobothrium’* cf. *cameroni* Rausch, 1969).

**Fig. 2:**
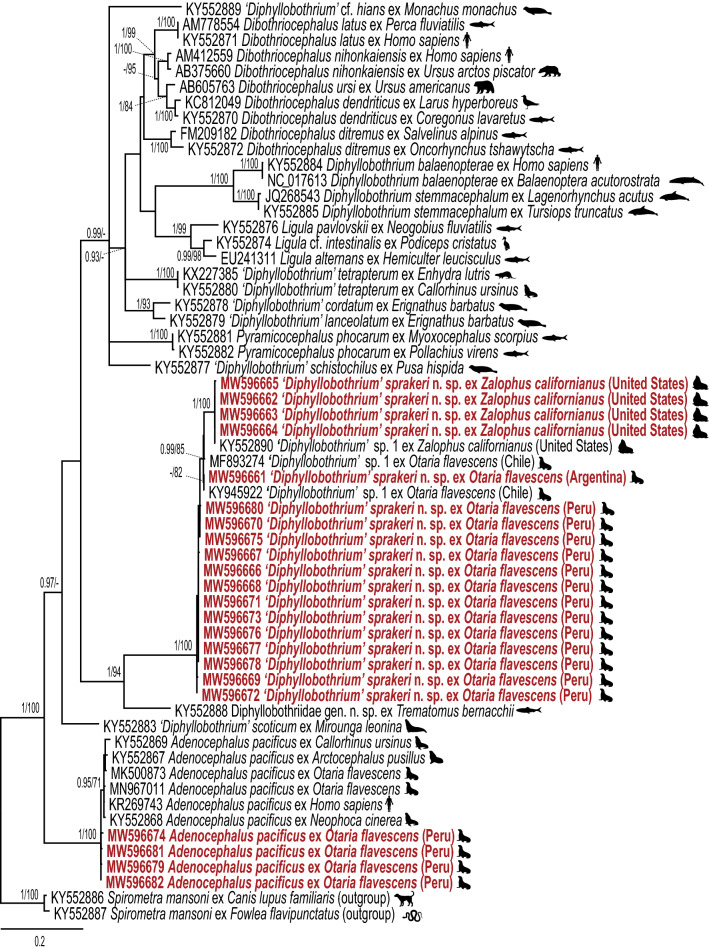
Bayesian analysis of the *cox*1 dataset for representatives of the family Diphyllobothriidae. Numbers represent posterior probabilities from BI analysis (> 0.95 shown only) followed by nodal supports from ML analysis (bootstrap values > 70% shown only). The newly generated sequences are indicated in red. GenBank accession numbers are shown before the species names. The scale bar indicates the expected number of substitutions per site

Both ML and BI analyses of the *cox*1 dataset (59 taxa) resulted in generally similar topologies (Fig. 2), but slightly differed from the recent phylogenetic study of Waeschenbach et al. [4]. Four newly generated sequences from isolates from CSLs and 14 novel sequences from SASLs (13 from Peru and 1 from Argentina) formed a strongly supported clade with three sequences identified as *'Diphyllobothrium'* sp. 1., including one sequence from an isolate from CSL (KY552890) and two from SASLs (MF893274 and KY945922) (Fig. 2). The genetic divergence between isolates from CSLs and SASLs in the *cox*1 gene was 2.87–3.07%. In the phylogenetic tree, the clade formed by our novel sequences + *'Diphyllobothrium'* sp. 1. appeared also as sister to the sequence of Diphyllobothriidae gen. sp. from *T. bernacchii* (KY552888). Genetic divergence between our newly generated sequences + *‘Diphyllobothrium’* sp. 1 and the isolate reported as Diphyllobothriidae gen. sp. ranged from 14.56–14.94%.

In contrast to the phylograms inferred for the *lsr*DNA dataset, our novel sequences + *'Diphyllobothrium'* sp. 1 + Diphyllobothriidae gen. sp. formed the sister group, although with low support, to an unresolved clade of diphyllobothriids (Fig. 2). The interspecific distances in the *cox*1 region of *'Diphyllobothrium'* spp. of pinnipeds ranged between 1.88% (*'Diphyllobothrium' tetrapterum* *vs* *'Diphyllobothrium' schistochilus*) to 14.30% (*'Diphyllobothrium' tetrapterum* *vs* *'Diphyllobothrium'* cf. *hians* (Diesing, 1850)). Finally, four newly generated partial sequences from isolates from SASL from Peru formed a strongly supported monophyletic lineage with the previously published sequences of *A. pacificus* (Fig. 2).

The phylogenetic trees inferred with the combined (*lsr*DNA + *cox*1) dataset (33 taxa) were similar to the topologies inferred for the *lsr*DNA and *cox*1 datasets (Additional file 5: Fig. S1). Our newly generated sequences formed a well-supported clade with sequences of *'Diphyllobothrium'* sp. 1 from CSL of California [4] and *'Diphyllobothrium'* sp. 1 from SASL of Chile [19].

In summary, both ML and BI analyses for the *lsr*DNA, *cox*1 and the combined datasets revealed that most of our newly generated sequences from isolates from CSLs and SASLs belong to an as yet undescribed species reported as *'Diphyllobothrium'* sp. 1 [4]. Genetic variation was detected between sequences from isolates of the putative new species from CSLs and SASLs. However, these values were somewhat lower, especially for *cox*1, than the interspecific variation between other *‘Diphyllobothrium’* species of pinnipeds included in our phylogenetic analyses. Therefore, we consider isolates of the putative new species of *'Diphyllobothrium'* from CSLs and SASLs from North and South America, respectively, to be conspecific. This new tapeworm species is described below.

**Family Diphyllobothriidae Lühe, 1910**

**Genus** ***Diphyllobothrium*** **Cobbold, 1858**

***Diphyllobothrium sprakeri*** **Hernández-Orts, Kuzmina, Gomez-Puerta & Kuchta n. sp.**

*Synonyms***:** *Diphyllobothrium* sp. 1 of Waeschenbach et al. [4], Hermosilla et al. [24] and Kuzmina et al. [18]

***Type-host*:** *Zalophus californianus* (Lesson) (Carnivora: Otariidae), California sea lion.

***Type-locality*:** Off central California (36°57'–38°32'N, 121°95–123°00'W), USA.

***Other host***: *Otaria flavescens* Shaw (Carnivora: Otariidae), South American sea lion.

***Other localities***: Los Angeles and San Diego, California, USA; Mexican waters (see below); Playa Unión (43°19′S, 65°03′W), Chubut, Argentina; beach of Bellavista (12°04′S, 77°07′W), Callao City, Callao, Peru.

***Site in host***: Small intestine.

***Prevalence in type-host***: 38% (15 out of 39 examined sea lions).

***Intensity in type-host***: 1–30 (average = 9.5) tapeworms per sea lion; most of the tapeworm specimens collected (> 70%) were in immature stage.

***Intensity in South American sea lions***: 1–17 immature tapeworms per host.

***Representative DNA sequences***: GenBank accession numbers: MW600337–MW600339 (*lsr*DNA) and MW596662–MW596665 (*cox*1) from *Z. californianus*: MW600336 (*lsr*DNA) and MW596661, MW596666–MW596673, MW596675–MW596678, MW596680 (*cox*1) from *O. flavescens*.

***Deposition of specimens***: Holotype (IPCAS C-765/1), seven slides of whole mounts and five slides of histological sections; one paratype (NMNH-USNM 1642475), 14 slides of whole mounts; one voucher (NMNH-USNM 1642476), eight slides of whole mounts; 10 vouchers (LEVE 986–990, 992–995, 997), two vouchers (PCMNH 4711, 4712) and one voucher (IPCAS C-765/2) immature worms from *O. flavescens* of Peru; three vouchers (IPCAS C-765/3), immature worms from *O. flavescens* of Argentina.

***ZooBank registration***: The Life Science Identifier (LSID) of the article is urn:lsid:zoobank.org:pub: 68C9D942-6D3A-4CF9-8732-4766EBA494DB. The LSID for the new name *Diphyllobothrium sprakeri* n. sp. is urn:lsid:zoobank.org:act: B95F5960-A589-4B34-BBE2-21E1D2EF368A.

***Etymology***: The species is named after Prof. Terry R. Spraker from the Colorado State University, Colorado, USA, for his valuable contribution to the collection of this species and studies of various groups of parasites of marine mammals.

### Description

**Fig. 3:**
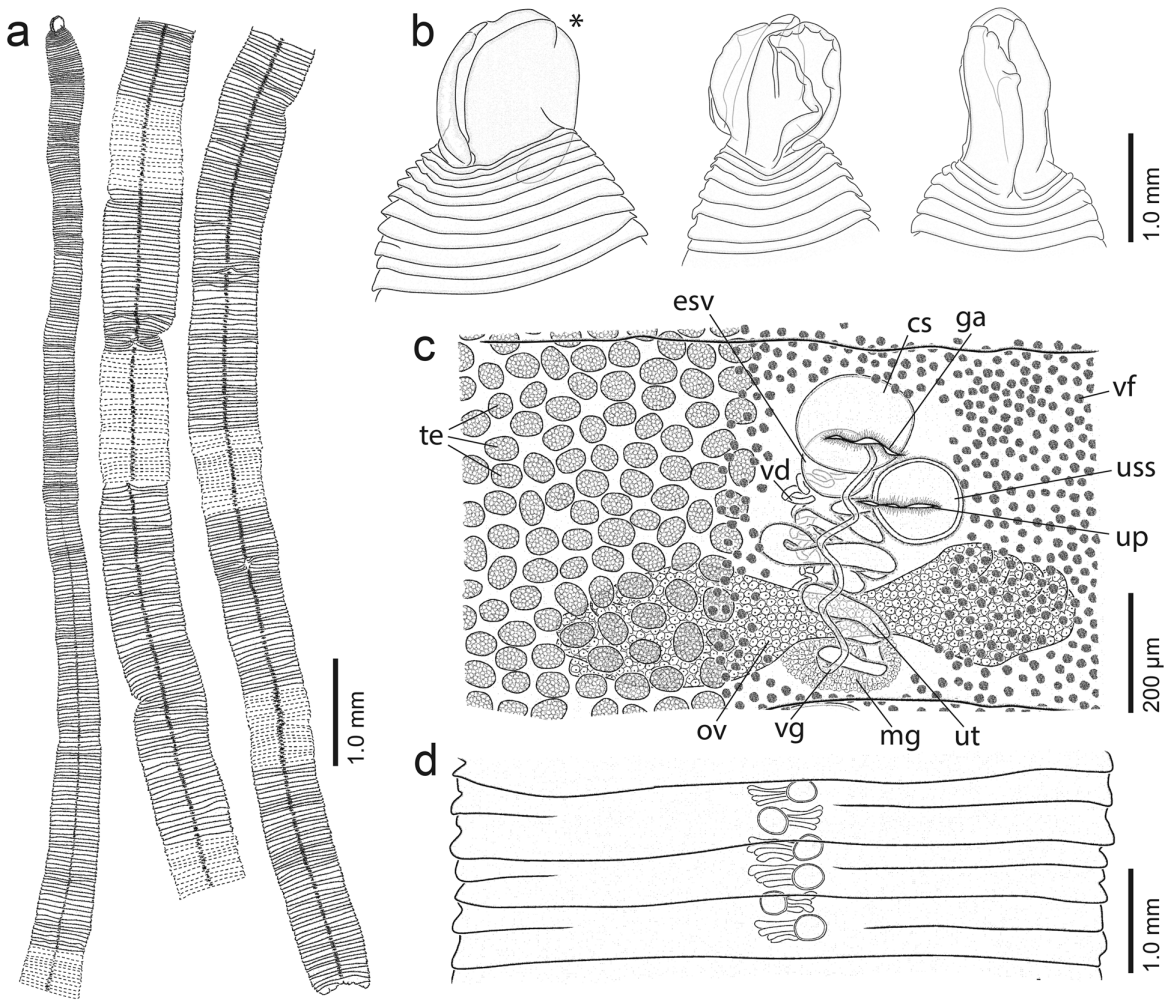
Line drawings of *‘Diphyllobothrium’ sprakeri* n. sp. from *Zalophus californianus*, California, USA. **a** Selected anterior, middle and posterior proglottids of holotype, ventral view. **b** Scoleces, dorsoventral view. Scolex of holotype marked with an asterisk. **c** Genitalia of mature proglottids of holotype, ventral view, vitelline follicles omitted on left side and testes on the right side of proglottid. **d** Schematic drawing of gravid proglottids of holotype showing the position of the sac-like structure of the uterus. *Abbreviations:* cs, cirrus sac; esv, external seminal vesicle; ga, genital atrium; mg, Mehlis' gland; ov, ovary; te, testes; up, uterine pore; uss, uterine sac-like structure; ut, uterus; vf, vitelline follicle; vg, vagina

**Fig. 4:**
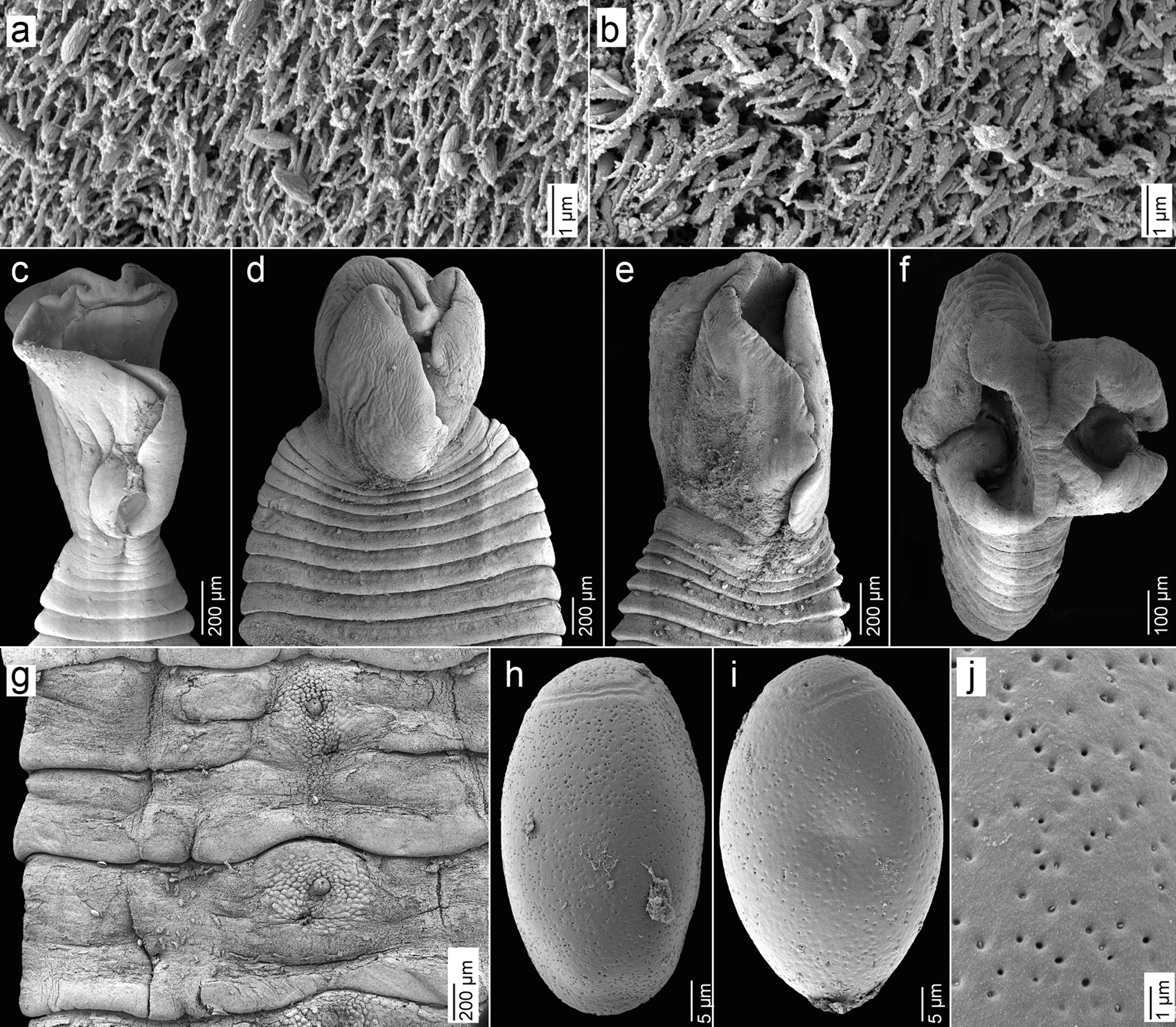
Scanning electron micrographs of *‘Diphyllobothrium’ sprakeri* n. sp. from *Zalophus californianus*, California, USA. **a** Capilliform filitriches on the lateral scolex surface.** b** Coniform spinitriches on the dorsoventral scolex surface. **c–e** Scoleces, dorsoventral view. **f** Scolex in apical view. **g** Ventral surface of gravid proglottids showing numerous papillae surrounding the genital atrium. **h–i** Eggs and surface of egg shell. **j** Detail of egg surface with pits

[Based on 20 specimens from CSLs. All tapeworms collected from SASL were immature specimens and were not included in the description]. Diphyllobothriidea, Diphyllobothriidae. Specimens anapolytic, long worms up to 2 m (holotype 56 cm; Fig. 3a) in length; maximum width ca. 8 mm. Immature worms may reach 1 m in length. Scolex surface covered with capilliform filitriches and coniform spinitriches (Fig. 4a, b). Microtriches on strobila surface not observed.

**Fig. 5:**
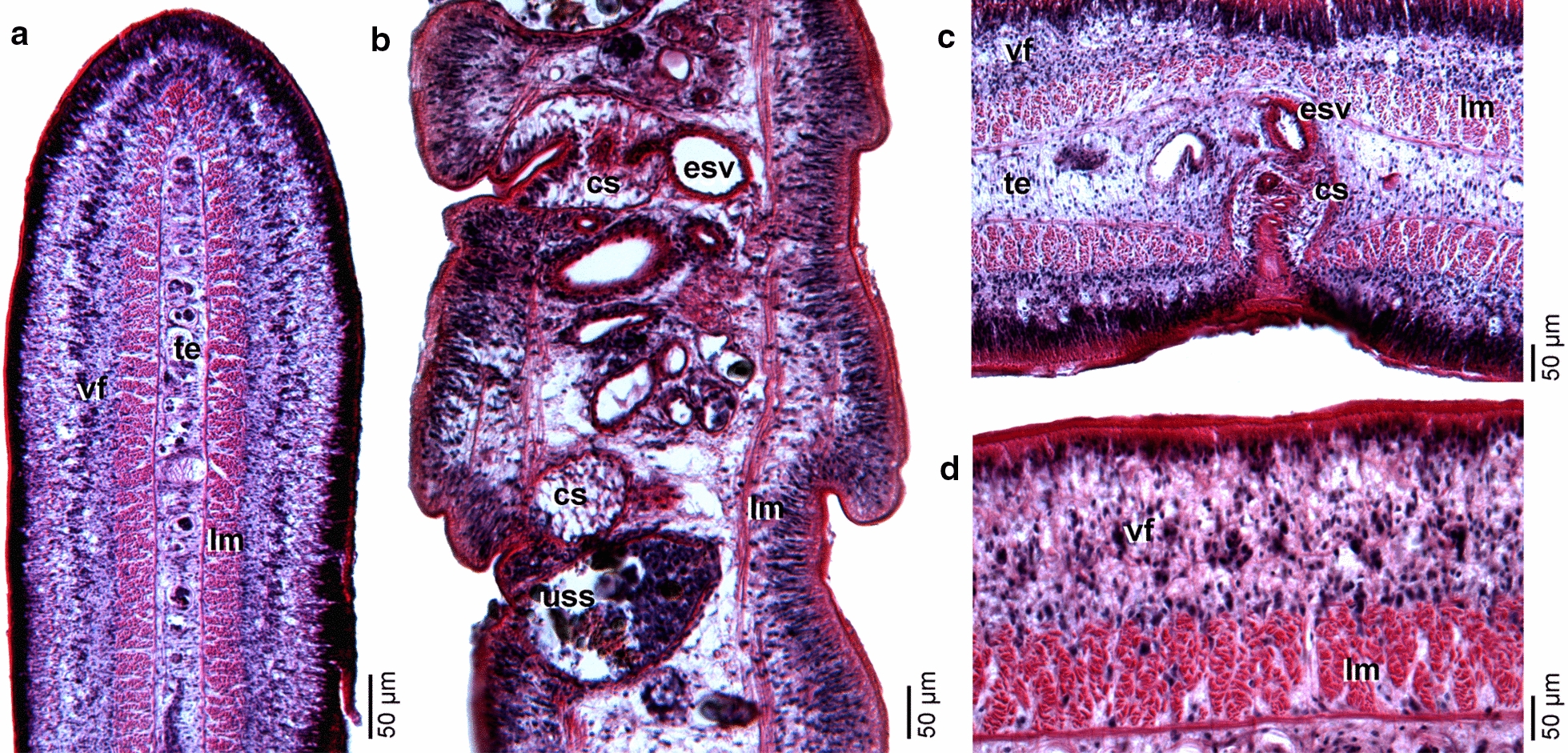
Photomicrographs of histological sections of holotype of *‘Diphyllobothrium’ sprakeri* n. sp. from *Zalophus californianus*, California, USA. **a** Gravid proglottid, cross section. **b** Gravid proglottids, sagittal section. **c** Detail of cirrus sac and genital atrium in gravid proglottid, cross section. **d** Detail of longitudinal musculature. *Abbreviations:* cs, cirrus sac; esv, external seminal vesicle; lm, longitudinal musculature; te, testes; uss, uterine sac-like structure; vf, vitelline follicle

Excretory system consists of cortical and medullary longitudinal canals extending throughout strobila; canals in cortex numerous, interspersed in layer of vitelline follicles or displaced towards tegument; 2 pairs of main excretory canals in medulla alongside median axis. Longitudinal musculature formed by muscle bundles, in layer surrounding transverse musculature (Fig. 5a,c,d); longitudinal muscle layer 51–100 (71.2 ± 18, *n* = 10) wide.

Scolex lanceolate, roundish or slightly elongated to triangular in dorsoventral view (Figs. 3b, 4c–e); 999–1484 (1207 ± 120, *n* = 15) long by 807–1275 (935 ± 132, *n* = 15) wide. Bothria with wide margins in lateral view, fan-like (Figs. 3b, 4c–e); bothrial margins fused anteriorly, leaving opening of varying degrees in middle and posterior parts (Figs. 3b, 4 c–f). Neck absent.

Proglottids craspedote, much wider than long, first eggs appear around 10 cm posterior to scolex; first proglottids posterior to scolex short, much wider than long, 51–188 (105.9 ± 37, *n* = 30) long by 821–1553 (1141 ± 214, *n* = 30) wide; proglottid width/length ratio 1:0.04–0.22 (0.1, *n* = 30). Mature proglottids, i.e. with spermatozoa in vas deferens, few, 1–3 in number (Fig. 3c), 281–401 (340 ± 42, *n* = 7) long by 3115–6202 (4451 ± 1177, *n* = 7) wide; proglottid width/length ratio 1:0.05–0.12 (0.08, *n* = 7). Gravid proglottids numerous, larger and wider, not enlarging, 287–736 (519 ± 130, *n* = 7) long by 2681–7890 (4712 ± 1921, *n* = 7) wide; proglottid width/length ratio 1:0.05–0.22 (0.14, *n* = 7).

Testes medullary, subspherical to oval, 44–70 (56 ± 6, *n* = 16) in diameter, 220–384 (325 ± 61, *n* = 7) in number, arranged in a single dorsoventral layer (Figs. 3c, 5a), form 2 lateral fields confluent at anterior and posterior margins of proglottid, not overlapping anterior uterine loops, absent in central area of cirrus sac, uterus and ovary (Fig. 3c). Vas deferens coiled, runs dorsally to uterus in midline to posterior border of external seminal vesicle (Fig. 3c). External seminal vesicle muscular, wall up to 27 thick, posterodorsal to cirrus sac, oval to almost elliptical in sagittal section (Figs. 3c, 5b), 58–122 (103 ± 19, *n* = 9) long by 38–120 (82 ± 21, *n* = 9) wide in dorsoventral view; length/width ratio 1:0.7–1 (0.8, *n* = 9). Cirrus sac thin-walled, wall up to 23 thick, larger than external seminal vesicle, oval in sagittal section (Fig. 5b), 142–286 (206 ± 40, *n* = 9) long by 92–159 (127 ± 18, *n* = 9) wide; length/width ratio 1:0.5–0.7 (0.6, *n* = 9). Cirrus sac opening in anterior region of genital atrium (Fig. 5b). Internal sperm duct thin-walled, strongly coiled; cirrus unarmed. Genital atrium ventral, median, pre-equatorial, situated 79–242 (201 ± 32, *n* = 7) from anterior margin of proglottid, representing 29–60% (40%) of proglottid length; surface area surrounding genital atrium covered with numerous papillae (Fig. 4g).

Ovary bilobed, situated dorsally near posteriormost margin of proglottid, 1029–1550 (1302 ± 167, *n* = 10) wide; ovarian lobes 135–233 (181 ± 30, *n* = 10) long, posteriorly enclosing Mehlis' gland (Fig. 3c). Vagina runs ventrally, 19–22 wide in cross sections (Fig. 5b). Female genital pore posterior to male genital opening in genital atrium (Fig. 5b). Vitelline follicles cortical, subspherical, numerous, 18–38 (29 ± 5, *n* = 18) in maximum diameter in dorsoventral view (Fig. 3c); area surrounding genital atrium devoid of vitelline follicles (Figs. 3c, 5c). Uterus tightly coiled, containing fully developed, but unembryonated eggs, forms bilateral uterine loops; uterine loops 3–4 (3, *n* = 5) in number on each side of mid-line (Fig. 3c). Terminal part of uterus enlarged, forming a thick-walled sac-like structure (Fig. 3c, d). Uterine pore situated 16–152 (75 ± 44, *n* = 7) from anterior margin of proglottid, representing 5–31% (21%) of proglottid length. Eggs oval, thick-walled, operculated (Fig. 4h, i), 59–75 (65 ± 3; *n* = 152) long by 40–47 (43 ± 2; *n* = 152) wide. Egg shell slightly pitted, with around 39–57 (*n* = 2) pits/10 µm^2^ (Fig. 4j).

### Remarks

The new species is placed in the genus *Diphyllobothrium* because of its typical shape of the scolex, composition of genital organs, absence of transverse papilla-like tegumental protuberances on the ventral surface of the proglottids and using pinnipeds as its definitive host (see [4]).

Kuchta and Scholz [3] reported 20 species of ‘*Diphyllobothrium*’ (*incertae sedis*) from the intestine of pinnipeds. Of these, *D. sprakeri* n. sp. can be distinguished from *D. archeri* (Leiper & Atkinson, 1914), *D. cameroni* Rausch, 1969, *D. cordatum* (Leuckart, 1863), *D. elegans* (Krabbe, 1865), *D. fayi* Rausch, 2005, *D. hians* (Diesing, 1850), *D. lanceolatum* (Krabbe, 1865), *D. lashleyi* (Leiper & Atkinson, 1914), *D. minutus* Andersen, 1987, *D. mobile* (Rennie & Reid, 1912), *D. phocarum* Delyamure, Kurochkin & Skryabin, 1964, *D. pseudowilsoni* Wojciechowska & Zdzitowiecki, 1995, *D. quadratum* (von Linstow, 1892), *D. rauschi* Andersen, 1987, *D. roemeri* (Zschokke, 1903), *D. schistochilos* (Germanos, 1895), *D. tetrapterum* (von Siebold, 1848) and *D. wilsoni* (Shipley, 1907) by having an enlarged thick-walled sac-like structure in the terminal part of the uterus in mature and gravid proglottids [41].

Recently, Hermosilla et al. [24] collected two headless tapeworms from fecal samples of SASLs in Chile and considered them a ‘*Diphyllobothrium scoticum*-like cestode.’ The morphological description of these tapeworms by Hermosilla et al. [24] is incomplete; however, they are similar to *D. sprakeri* n. sp. in having a thick-walled sac-like structure in the terminal part of the uterus, longitudinal muscle layer wide (51–100 *vs* 100) and egg size (54–61 × 38–44 *vs* 59–75 × 40–47). The new species slightly differs from the the ‘*Diphyllobothrium scoticum*-like cestode’ by having smaller gravid proglottids (287–736 × 2681–7890 *vs* 825–1385 × 7077–7418) and number of uterine loops (3–4 *vs* 5–6). However, the size of diphyllobothriids and the number of uterine loops have a limited taxonomic value in distinguishing species (see [2, 5] and references therein). Our phylogenetic analyses confirmed that specimens of *D. sprakeri* n. sp. recovered from CSLs are conspecific with those from SASLs from Chile (Figs. 1, 2).

Two species of ‘*Diphyllobothrium*’ described from Antarctic phocids, i.e. *D. scoticum* and *D. lobodoni* Yurakhno & Maltsev, 1994, are similar to *D. sprakeri* n. sp. in having a thick-walled sac-like structure in the terminal part of the uterus [22, 42–46]. The new species can be distinguished from *D. scoticum* and *D. lobodoni* by its smaller scolex (< 1484 *vs* > 1800 and > 1900, respectively), absence of a neck, lower number of uterine loops (3–6 *vs* 5–17 and 7–22, respectively) and somewhat smaller eggs (59–75 × 39–47 *vs* 60–100 × 42–53 and 71–74 × 51–53, respectively) (minimum and maximum range for biometrical data for *D. scoticum* estimated from all available descriptions; see Table 1 for details). ‘*Diphyllobothrium*’ *sprakeri* n. sp. can be further differentiated from *D. scoticum* and *D. lobodoni* in the natural definitive hosts and the disparate geographical distribution (otariids from temperate waters of North and South America *vs* Antarctic phocids) (see below).

**Table 1:** Comparison of selected biometrical data among *‘Diphyllobothrium’ sprakeri* n. sp., *‘Diphyllobothrium’ scoticum* and *‘Diphyllobothrium’ lobodoni*. The incomplete description of *D. scoticum* from leopard seals from Macquarie Island, Antarctica, by Johnston [44] is not included. Measurements in micrometers, unless otherwise stated

Species	*D. sprakeri* n. sp.	*D. sprakeri* n. sp.1^a^	*D. scoticum*^c^	*D. scoticum*^d^
Host	*Zalophus californianus* (Lesson)	*Otaria flavescens* Shaw	*Hydrurga leptonyx* (Blainville)	*H. leptonyx*
Locality	California, USA	Los Lagos Region, Chile	Antarctica	Antarctica
GenBank accession no	MW600337–MW600339 (*lsr*DNA); MW596661, MW596666–MW596673, MW596675–MW596678, MW596680 (*cox*1)	KY945917 (*lsr*DNA); KY945922, MF893274 (*cox*1)	–	–
Reference	Present study	Hermosilla et al. [24, 25]	Rennie and Reid [42]	Fuhrmann [43]^d^
Strobila length (cm)	< 200 × 0.8	> 50	13.3–29	13.3
Scolex	999–1484 × 807–1275	–	1800–2500 × 1500	1800–3000 × 700–2500
Neck	Absent	–	Short	Absent
Gravid proglottid (cm)	0.03–0.07 × 0.27–0.79	0.08–0.14 × 0.71–0.74	0.09 × 0.15	0.12 × 0.55
Testes diameter or size	44–70	150^b^	69–87	100–160
Ovary width	1029–1550	–	–	–
Number of uterine loops	3–4	5–6	> 4	5–7
Uterine sac-like structure	Present	Present	Present	Present
Egg size	59–75 × 40–47	54–61 × 39–44	70–100 × 43–51	64–80 × 44–48

Species	*D. scoticum*	*D. scoticum*	*D. lobodoni*	*D. scoticum*
Host	*H. leptonyx*	*H. leptonyx*	*Lobodon carcinophagus* (Hombron & Jacquinot)	*H. leptonyx*
Locality	Debenham Islands, Antarctica	Balleny Islands, D'Urville Sea, Antarctica	Balleny Islands, D'Urville Sea, Antarctica	King George Island, South Shetland Islands
GenBank accession no	–	–	–	–
Reference	Markowski [22]	Yurakhno and Maltsev [45]	Yurakhno and Maltsev [45]	Wojciechowska and Zdzitowiecki [46]
Strobila length (cm)	52–130	5.6–42	44.2–240	16–150
Scolex	3500 × 2000	1900–3500 (length)	1900–2870 (length)	2200–4600 × 1100–2300
Neck length	495	370–1500	910–1290	Present
Gravid proglottid (cm)	0.50–0.80 × 1.50–1.80	–	0.35 × 1.35	0.16–0.8 × 0.13–0.6
Testes diameter or size	150–210 × 150	69–193	100–220	67–150 × 47–1400
Ovary width	–	–	4900 × 5100	–
Number of uterine loops	5–12	5–17	7–22	–
Uterine sac-like structure	Present	Present	Present	Present
Egg size	76–79 × 56	68–76 × 50–53	71–74 × 51–53	60–85 × 42–56

^a^Specimens without scoleces referred to as ‘*Diphyllobothrium scoticum*-like cestode’

^b^Testes measured in transverse section

^c^Metrical data on *Dibothriocephalus pygoscelis* Rennie & Reid, 1912, which is considered a junior synonym of *D. scoticum* (see Johnston [44], Markowski [22]), is included

^d^Fuhrmann [43] examined the holotype of *D. scoticum*

Our molecular analyses reported immature specimens of *A. pacificus* and *D. sprakeri* n. sp. in the intestine of a SASL from Peru. Immature specimens of both species are morphologically indistinguishable. Adult specimens of *A. pacificus* can be distinguished from the new species by the presence of the papilla-like protuberances anterior to the male gonopore [2] and the absence of a thick-walled sac-like structure in the terminal part of the uterus. To our knowledge, this is the first confirmed report of coinfection of two species of diphyllobothriid tapeworms in a single otariid from the Southern Hemisphere. Voucher specimens of *A. pacificus* collected in this study are deposited in the Laboratory of Epidemiology and Veterinary Economics (LEVE 910, 913, 917, 923, 925), Lima, Peru.

The voucher material identified as ‘*Diphyllobothrium latum*’ from CSLs off Mexico and the USA deposited in London (NHML) was substantially decomposed. These specimens were immature without developed genital organs crucial for identification. However, *D. sprakeri* n. sp. is similar to these specimens in the shape of the scolex and the absence of a neck. ‘*Diphyllobothrium*’ *sprakeri* n. sp. has been the only species of ‘*Diphyllobothrium*’ reported from the intestine of CSLs ([18], present study). Based on this evidence, the voucher material deposited in London is tentatively conspecific with the new species. One voucher specimen deposited in London (NHML 1980.6.3.189) was collected from a CSL stranded on the Mexican coast. In Mexico, CSLs are distributed along the east and west coasts of the Baja California Peninsula [47]. Therefore, this peninsula may represent an additional locality for *D. sprakeri* n. sp.

## Discussion

The taxonomy of diphyllobothriids is insufficiently resolved. Identification of individual species is complicated because of their uniform strobilar morphology, the high amount of intraspecific and intraindividual variation for most morphological characters and incomplete original descriptions [3, 8].

Diphyllobothriids of pinnipeds have been revised by the present authors for more than 10 years based on detailed morphological examination of well-fixed material combined with molecular data (see [2, 4–6, 8, 40]). Our previous studies suggested that otariids are only definitive hosts of three diphyllobothriid species: *A. pacificus*, widely distributed in both hemispheres, and *D. tetrapterum* and *Pyramicocephalus phocarum* (Fabricius, 1780), limited to the Northern Hemisphere [2, 5, 8]. However, a revision of the metazoan parasites of CSLs recognized a new undescribed diphyllobothriid tapeworm which is different from these three species [4, 5, 18]. This species showed a uterine sac-like structure in mature and gravid proglottids, which is an uncommon character in diphyllobothriids. ‘*Diphyllobothrium*’ *sprakeri* n. sp. is the first diphyllobothriid species described from CSLs and, with *A. pacificus*, the second valid species of otariids from the Southern Hemisphere.

A uterine sac-like structure was described in *D*. *scoticum* from leopard seals *Hydrurga leptonyx* (Blainville) from several localities in Antarctica [22, 42–46]. This tapeworm species has also been reported in Weddell seals *Leptonychotes weddellii* (Lesson) in Antarctica and recently confirmed using molecular markers from southern elephant seals *Mirounga leonina* (L.) from Macquarie Island (Southwestern Pacific Ocean) [8, 46, 48].

Yurahkno and Maltsev [45] described *D. lobodoni* from the intestine of crabeater seals *Lobodon carcinophagus* (Hombron & Jacquinot) from Antarctica. This diphyllobothriid species differs from *D. scoticum* by the size of the strobila, scolex, neck and uterine sac-like structure, the shape of proglottids, thickness of the tegument and muscle layer, number of testes and the position of the cirrus sac and external seminal vesicle. However, the size of the strobila and scolex and number of testes and other structures may depend on the fixation methods, host species, its size, physiological state or intensity of infection (see [5], and references therein) and are not suitable characters in species delimitation [2, 3]. Moreover, most of the used discriminant characteristics of *D. lobodoni* overlap with those of *D. scoticum* reported from leopard seals (type-host) by other authors (Table 1). Further studies of the type material and molecular data from the type-host of *D. lobodoni* are necessary to confirm the validity and systematic position of this species.

Our study suggests that *D. sprakeri* n. sp. has a wide geographical distribution in both hemispheres, including the Pacific and Atlantic Oceans, and infects at least two otariid species. The distribution of *D. sprakeri* n. sp. in the Northern Hemisphere is limited to the Pacific coast of California, USA, and Baja California, Mexico. Interestingly, our new species has not been recorded in the North Pacific coast, where the diversity of diphyllobothriid tapeworms from otariids has been comprehensively evaluated in recent years [2, 5, 49, 50]. In the Southern Hemisphere, *D. sprakeri* n. sp. is more widely distributed, occurring in temperate waters of the Pacific coast of South America (Peru and Chile) and the Southwest Atlantic along the Patagonian coast of Argentina.

The life cycle of *D. sprakeri* n. sp. probably includes marine fishes as the second intermediate hosts. Recently, Mondragón Martínez [51] reported plerocercoids of *A. pacificus* and an unidentified species of *Diphyllobothrium* in marine fishes from Peru based on partial *cox*1 sequences. According to the phylogenetic analysis of Mondragón Martínez [51], unidentified diphyllobothriid plerocercoids, collected from anchoveta *Engraulis ringens* Jenyns and Pacific jack mackerel *Trachurus symmetricus* (Ayres), formed a clade sister to *A. pacificus* and *Diphyllobothrium* spp. Unfortunately, partial *cox*1 sequences of these diphyllobothriid plerocercoids are not available in the GenBank dataset. These plerocercoids probably belong to *D. sprakeri* n. sp.; however, sequences generated from these plerocercoids need to be analyzed in a more robust phylogenetic context for reliable species identification.

The Pacific broad tapeworm *Adenocephalus pacificus* is considered the most important causative agent of diphyllobothriosis among humans in South America [52]. Diphyllobothriosis caused by this species has been reported predominantly in Peru, where human infections are associated with the habits of consuming raw or undercooked marine fishes [23, 52]. Our new species may be also causative agent of human fish-borne disease in the Pacific coast of South America, but not yet recognized and certainly misidentified as *A. pacificus*. Molecular-based diagnoses represent the most reliable tool to identify clinical samples of diphyllobothriid tapeworms [53], especially because clinical samples or immature specimens of *A. pacificus* and *D. sprakeri* n. sp. could be morphologically indistinguishable.

## Supplementary Information

**Additional file 1: Table S1.** List of taxa used in the phylogenetic analyses.

**Additional file 2:** ***lsr*DNA_alignment.** Trimmed *lsr*DNA alignment (34 taxa; 1574 bp).

**Additional file 3: cox1_alignment.** Trimmed *cox*1 alignment (59 taxa; 1571 bp).

**Additional file 4:** ***lsr*DNA_cox1_alignment.** Combined *lsr*DNA + *cox*1 alignment (33 taxa; 3145 bp).

**Additional file 5: Figure S1.** Bayesian analysis for the combined (*lsr*DNA + *cox*1) alignment. Numbers represent posterior probabilities from BI analysis (> 0.95 shown only) followed by nodal supports from ML analysis (bootstrap values > 70% shown only). The newly generated sequences are indicated in red. The scale bar indicates the expected number of substitutions per site.

## Data Availability

The type- and voucher material is deposited in the Helminthological Collection of the Institute of Parasitology, Česke Budějovice, Czech Republic, the National Museum of Natural History of the Smithsonian Institution, Washington DC, USA, the Parasite Collection of the Museum of Natural History, National University of San Marcos, Lima, Peru, and the Parasite Collection of the Laboratory of Epidemiology and Veterinary Economics, National University of San Marcos, Lima, Peru (see Results for accession numbers). DNA sequences generated in this study were deposited in the GenBank database (see Additional file 1: Table S1 for details). Alignments used in this study are included as additional files.

## Abbreviations

BI: Bayesian inference
*cox*1: Cytochrome *c* oxidase subunit 1 gene
cs: Cirrus sac
CSL: California sea lion
esv: External seminal vesicle
ga: Genital atrium
IPCAS: Helminthological Collection of the Institute of Parasitology
LEVE: Parasite Collection of the Laboratory of Epidemiology and Veterinary Economics
*lsr*DNA: Large ribosomal subunit nuclear ribosomal RNA gene
mg: Mehlis' gland
ML: Maximum likelihood
NMNH-USNM: National Museum of Natural History of the Smithsonian Institution
ov: Ovary
PCMNH: Parasite Collection of the Museum of Natural History
SASL: South American sea lion
te: Testes
up: Uterine pore
uss: Uterine sac-like structure
ut: Uterus
vf: Vitelline follicle
vg: Vagina
