# Supplementary material for: Diphyllobothrium sprakeri n. sp. (Cestoda: Diphyllobothriidae): a hidden broad tapeworm from sea lions off North and South America
Source: Parasit Vectors. 2021 Apr 22;14:219. doi: 10.1186/s13071-021-04661-1 (PMC8063393; doi:10.1186/s13071-021-04661-1)
Supplement: Supplementary file 1 — Additional file 1: Table S1. List of taxa used in the phylogenetic analyses. [file 13071_2021_4661_MOESM1_ESM.docx]

**Supplementary Table S1** List of taxa used in this study, their hosts, localities and GenBank accession numbers. Sequences generated in this study are indicated in bold

| **Species** | **Host** | **Locality** | **GenBank accession number** | |
| --- | --- | --- | --- | --- |
|  |  |  | **lsrDNA** | ***cox*1** |
| *Adenocephalus pacificus* Nybelin, 1931 | *Arctocephalus pusillus* (Schreber) | Victoria, Australia | KY552808 | KY552867 |
|  | *Callorhinus ursinus* (L.) | Alaska, USA | KY552810 | KY552869 |
|  | *Homo sapiens* L. | Lima, Peru | DQ925327 | KR269743 |
|  | *Otaria flavescens* Shaw | Chile | – | MN967011 |
|  |  | Peru | – | MK500873 |
|  |  | Callao, Peru | – | **MW596674, MW596679, MW596681, MW596682** |
|  | *Neophoca cinerea* (Péron) | South Australia, Australia | KY552809 | KY552868 |
| *Dibothriocephalus dendriticus* (Nitzsch, 1824) | *Coregonus lavaretus* (L.) | Loch Doyne, Scotland | KY552812 | KY552870 |
|  | *Larus hyperboreus* Gunnerus | Kansas, USA | KY552814 | KC812049 |
| *Dibothriocephalus ditremus* (Creplin, 1825) | *Salvelinus alpinus* (L.) | Loch Doyne, Scotland | KY552813 | FM209182 |
|  | *Oncorhynchus tshawytscha* (Walbaum) | Oregon, USA | KY552815 | KY552872 |
| *Dibothriocephalus latus* (Linnaeus, 1758) | *Perca fluviatilis* L. | Como Lake, Italy | KY552816 | AM778554 |
|  | *H. sapiens* | Manitoba, Canada | KY552817 | KY552871 |
| *Dibothriocephalus nihonkaiensis* (Yamane, Kamo, Bylund & Wikgren, 1986) | *H. sapiens* | Geneva, Switzerland | – | AM412559 |
|  | *Ursus arctos piscator* (Bergman) | Kamchatka, Russia | – | AB375660 |
| *Dibothriocephalus ursi* (Rausch, 1954) | *Ursus americanus* Pallas | Alaska, USA | – | AB605763 |
| *Diphyllobothrium balaenopterae* (Lönnberg, 1892) | *H. sapiens* | Ishikawa, Japan | KY552824 | KY552884 |
|  | *Balaenoptera acutorostrata* Lacépède | North Pacific Ocean | – | NC_017613 |
| *‘Diphyllobothrium’* cf. *cameroni* Rausch, 1969 | *Neomonachus schauinslandi* (Matschie) | Hawaii, USA | KY552831 | – |
| *‘**Diphyllobothrium’ cordatum* (Leuckart, 1863) | *Erignathus barbatus* (Erxleben) | Alabama, USA | KY552822 | KY552878 |
| *‘Diphyllobothrium’* cf. *hians* (Diesing. 1850) | *Monachus monachus* (Hermann) | Mediterranean Sea | – | KY552889 |
| *‘**Diphyllobothrium’ schistochilus* (Germanos, 1895) | *Pusa hispida* (Schreber) | Svalbard, Norway | KY552821 | KY552877 |
| *‘Diphyllobothrium’ scoticum* (Rennie & Reid, 1912) | *Mirounga leonina* (L.) | Tasmania, Australia | KY552811 | KY552883 |
| *‘**Diphyllobothrium’ tetrapterum* (von Siebold, 1848) | *C. ursinus* | Alaska, USA | KY552826 | KY552880 |
|  | *Enhydra lutris* (Linnaeus) | California, USA | KX227386 | KX227385 |
| *‘Diphyllobothrium’ lanceolatum* (Krabbe, 1865) | *E. barbatus* | Alabama, USA | KY552823 | KY552879 |
| *Diphyllobothrium stemmacephalum* Cobbold, 1858 | *Lagenorhynchus acutus* (Gray) | Massachusetts, USA | AF286943 | JQ268543 |
|  | *Tursiops truncatus* (Montagu) | Mississippi, USA | KY552825 | KY552885 |
|  |  |  |  |  |
| *‘Diphyllobothrium’ sprakeri* n. sp. | *O. flavescens* | Callao Province, Peru | – | **MW596666-MW596673, MW596675**–**MW596678, MW596680** |
|  |  | Río Negro, Argentina | **MW600336** | **MW596661** |
|  | *Zalophus californianus* (Lesson) | California, USA | **MW600337**–**MW600339** | **MW596662**–**MW596665** |
| *‘Diphyllobothrium’* sp. 1 | *O. flavescens* | Aysén, Chile | KY945917 | KY945922; MF893274 |
|  | *Z. californianus* | California, USA | KY552829 | KY552890 |
| Diphyllobothriidae gen. sp. | *Trematomus bernacchii* Boulenger | Antarctica | KY552830 | KY552888 |
| *Ligula alternans* Rudolphi, 1810 | *Hemiculter leucisculus* (Basilewsky) | Far East, Russia | DQ925325 | EU241311 |
| *Ligula* cf. *intestinalis* (L.) | *Podiceps cristatus* (L.) | Moravia, Czech Republic | KY552819 | KY552874 |
| *Ligula pavlovskii* Dubinina, 1959 | *Neogobius fluviatilis* (Pallas) | Black Sea, Ukraine | KY552820 | KY552876 |
| *Pyramicocephalus phocarum* (Fabricius, 1780) | *Myoxocephalus scorpius* (L.) | Svalbard, Norway | KY552827 | KY552881 |
|  | *Pollachius virens* (L.) | Svalbard, Norway | KY552828 | KY552882 |
| Outgroup |  |  |  |  |
| *Spirometra mansoni* Cobbold (1883) | *Canis lupus familiaris* L. | Victoria, Australia | KY552835 | KY552886 |
|  | *Fowlea flavipunctatus* (Hallowell) | Vinh, Vietnam | KY552836 | KY552887 |
