# Supplementary figures and images for: Diphyllobothrium sprakeri n. sp. (Cestoda: Diphyllobothriidae): a hidden broad tapeworm from sea lions off North and South America
Source: Parasit Vectors. 2021 Apr 22;14:219. doi: 10.1186/s13071-021-04661-1 (PMC8063393; doi:10.1186/s13071-021-04661-1)

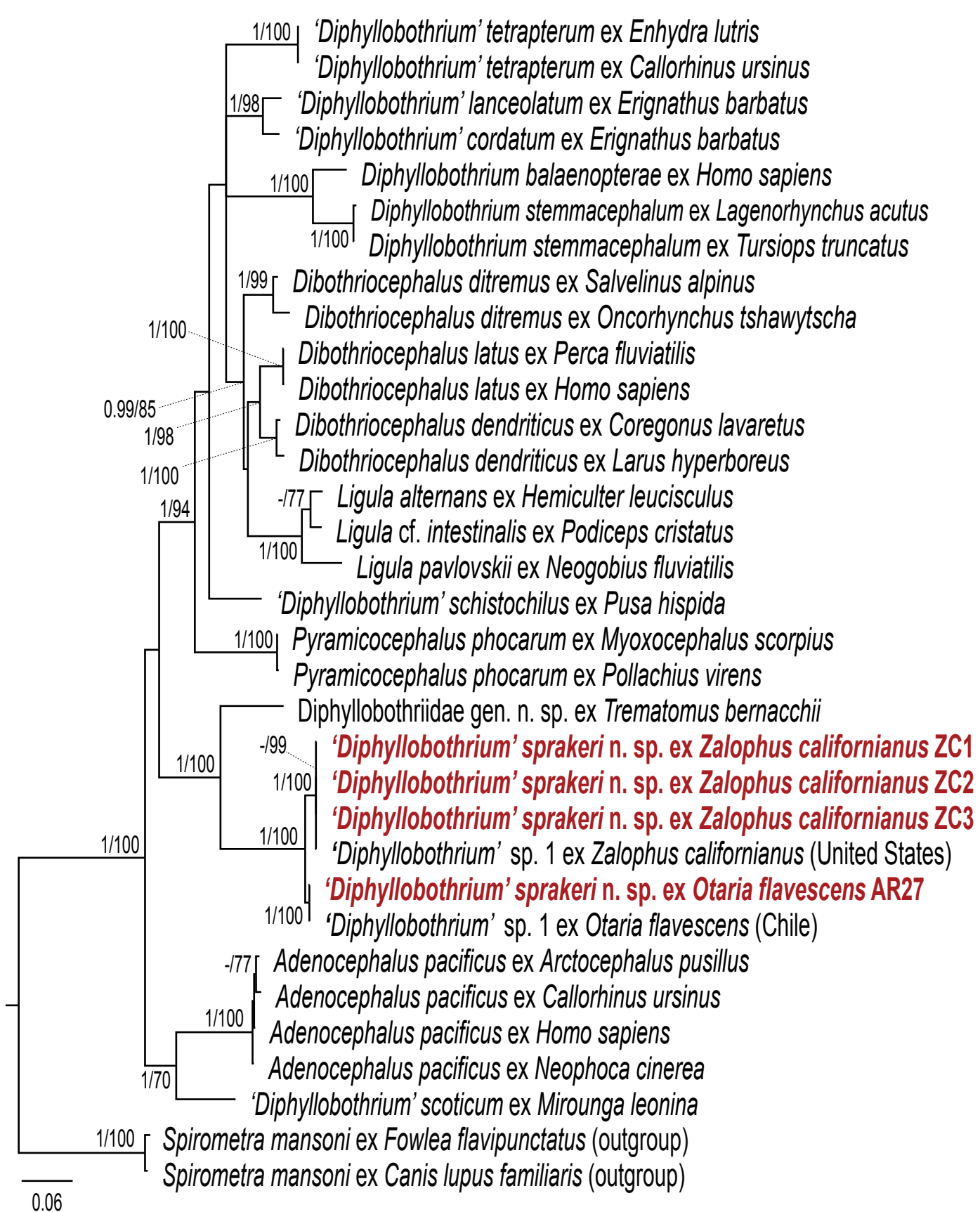

Supplement: Supplementary file 5 — Additional file 5: Figure S1. Bayesian analysis for the combined (lsrDNA + cox1) alignment. Numbers represent posterior probabilities from BI analysis (> 0.95 shown only) followed by nodal supports from ML analysis (bootstrap values > 70% shown only). The newly generated sequences are indicated in red. The scale bar indicates the expected number of substitutions per site. [file 13071_2021_4661_MOESM5_ESM.pdf]
